# COHORT DIFFERENCES IN SLEEP ENVIRONMENT, BEHAVIORS, AND CONCERNS: DESCRIPTIVE ANALYSES USING MY SLEEP SCRIPT APP

**DOI:** 10.1093/geroni/igz038.1942

**Published:** 2019-11-08

**Authors:** Christina Pierpaoli Parker, Rachel Salas, Charlene Gamaldo, Alyssa Gamaldo, Peter Dziedzic

**Affiliations:** 1 University of Alabama, Birmingham, Alabama, United States; 2 Johns Hopkins Medicine, Baltimore, Maryland, United States; 3 Johns Hopkins Medicine, Johns Hopkins Sleep Disorders Center, Maryland, United States; 4 Penn State College of Health & Human Development, Assistant Professor, Human Development and Family Studies, Penn State, Pennsylvania, United States; 5 Johns Hopkins University School of Medicine, Director of The Center of mHealth and Innovations, United States

## Abstract

Behavioral and environmental factors influence sleep outcomes. However, we understand little about how enviro-behavioral sleep hygiene practices and related sleep concerns vary across age cohorts. Using data from My Sleep Script, an app-based diagnostic checklist for identifying at risk patients, we described cohort differences in sleep hygiene and new sleep disturbances. 323 adults (46.6% female, 63.9% Caucasian) reported basic demographic, health, sleep, as well as enviro-behavioral data using the Epworth Sleepiness Scale (ESS), Pittsburg Sleep Quality Index (PSQI), and the Johns Hopkins Sleep Environment Instrument. We partitioned participants into four cohorts corresponding to birth year: The Silent Generation (N = 48, 14.9%), Baby Boomers (N = 124, 38.4%), Generation Xers (n = 109, 33.7%), and Millennials (N = 42, 13.0%). Spearman correlations described linkages among environment, behaviors, and sleep outcomes; a chi-square analysis, cohort differences in new sleep concerns. Having weapons, music players, lights, pets, and a disruptive sleep surface in the environment correlated with worse sleep quality. Eating, exercising, working, and sexual activity one hour before bed also correlated with worse sleep quality. Sleeping with pets, electronics, and on a disruptive surface correlated with lower sleep duration. Regarding cohort, we observed significant generational differences in new snoring and sleepiness complaints. Results confirm associations of suboptimal sleep hygiene with poor sleep outcomes and provide insights into their generational differences, warranting additional investigation.

